# TREC dynamics as a biomarker of naive T-cell homeostasis in traumatic brain injury: a longitudinal analysis

**DOI:** 10.3389/fmed.2026.1775886

**Published:** 2026-03-12

**Authors:** Darya A. Kashatnikova, Alesya S. Gracheva, Ekaterina V. Kalinina, Vladislav E. Zakharchenko, Tatyana N. Krylova, Maryam B. Khadzhieva, Sergey S. Larin, Artem N. Kuzovlev, Lyubov E. Salnikova

**Affiliations:** 1 Vavilov Institute of General Genetics, Russian Academy of Sciences, Moscow, Russia; 2 Lopukhin Federal Research and Clinical Center of Physical-Chemical Medicine of Federal Medical Biological Agency, Moscow, Russia; 3 Federal Research and Clinical Center of Intensive Care Medicine and Rehabilitology, Moscow, Russia; 4 National Research Center of Pediatric Hematology, Oncology and Immunology, Moscow, Russia

**Keywords:** age, composite ‘severity/inflammation’ index, linear mixed-effects models, naive T-cell homeostasis, TBI, TREC/KREC dynamics

## Abstract

Post-traumatic immunosuppression complicates recovery from traumatic brain injury (TBI), increasing susceptibility to infection. Reliable biomarkers to assess immune status are required. We investigated the dynamics of T-cell receptor excision circles (TREC) and B-cell K-deleting recombination excision circles (KREC) as potential markers of immune homeostasis in TBI patients. In this observational study, 51 patients with moderate-to-severe TBI were enrolled. Serial peripheral blood samples were collected for the purpose of quantifying TREC and KREC levels using real-time PCR. Linear mixed-effects models (LMMs) were employed to analyze the longitudinal dynamics and identify clinical predictors. Principal Component Analysis (PCA) was applied to construct a composite severity/inflammation index, and the final model was validated using a cluster bootstrap procedure. In most patients, the baseline TREC level was close to or below the lower age-matched norms. TREC and KREC levels fluctuated greatly, with non-monotonic changes and multi-fold variations. Three patients with decreasing TREC dynamics subsequently succumbed to sepsis. Lower TREC levels were robustly associated with older age and a higher severity/inflammation index (*p* < 0.001), while KREC dynamics remained independent of the examined clinical and inflammatory parameters. TREC levels restoration was synchronous with neurological improvement (rising GCS) and the resolution of organ dysfunction (declining SOFA). By offering a window into a key, potentially modifiable biological mechanism underlying patient vulnerability, TREC analysis represents a promising new approach for risk stratification and the development of future immunotherapeutic strategies in neurocritical care.

## 1 Introduction

Traumatic brain injury (TBI) remains a leading cause of mortality and long-term disability worldwide, particularly among young and working-age individuals (1). Beyond the primary neural damage, secondary complications significantly contribute to patient outcomes. Among these, infections such as pneumonia and sepsis are particularly noteworthy, and their development is closely linked to post-traumatic immunosuppression—a phenomenon characterized by the immune system’s reduced capacity to respond adequately to pathogens (2–4).

Existing clinical tools, such as the Sequential Organ Failure Assessment (SOFA) scale, are the standard for assessing the physiological severity and organ dysfunction in critically ill patients (5, 6). While SOFA provides a holistic assessment of patient condition, it does not incorporate direct measures of the functional state of the adaptive immune system. Consequently, the identification and validation of reliable biomarkers for monitoring immune status and restoring homeostasis in TBI patients is a critical objective in modern neurocritical care (7–9).

Potential candidates for such markers are circular DNA molecules formed during lymphocyte maturation: T-cell receptor excision circles (TRECs) and B-cell kappa-deleting recombination excision circles (KRECs). TREC levels in peripheral blood reflect the production of new T-lymphocytes in the thymus (thymopoiesis), while KREC levels reflect the production of B-lymphocytes in the bone marrow (B-lymphopoiesis) (10, 11). Age- and gender-related differences in TREC and KREC levels in adults are widely discussed. While TREC levels consistently decrease with age (12, 13), such changes are generally not observed for KRECs in adult populations (14). Some studies report higher TREC levels in women than in men (15, 16), though this is not a universal finding (17). Reduced TREC levels have been associated with a range of chronic conditions, particularly congenital and acquired immunodeficiencies (18, 19), autoimmune diseases (20), cardiovascular diseases (21, 22), and renal failure (23). Furthermore, the prognostic value of TREC levels in nosocomial and community-acquired infections (24, 25), especially COVID-19, is an area of active investigation (26–28).

Despite their established role as markers of immune function and their prognostic significance in various diseases, the dynamics of TREC and KREC have not been studied in the context of TBI. Our study aimed to examine the dynamics of TREC and KREC levels in patients with moderate-to-severe traumatic brain injury (TBI) during their hospital stays at a neuro rehabilitation facility. We sought to identify clinical and laboratory factors associated with changes in these indicators and determine if immune restoration aligns with clinical stabilization and neurological recovery.

## 2 Materials and methods

### 2.1 Study design and patient characteristics

This study enrolled 51 patients (41 men, 10 women) between the ages of 18 and 60. All participants suffered from moderate-to-severe traumatic brain injury (TBI) and were admitted for treatment and rehabilitation at the Federal Research and Clinical Center of Intensive Care Medicine and Rehabilitology (FRCC ICMR). The cohort comprised 38 patients from a previously described cohort (29, 30), with an additional 13 patients prospectively enrolled using the same inclusion criteria. The median time from injury to admission at the FRCC ICMR was 30 days (IQR 16–69).

Patients were excluded if they met any of the following criteria: age under 18 years; a history of mental or neurological diseases (e.g., stroke, CNS tumors) that could impact rehabilitation outcomes; developmental disorders; hereditary neurological diseases; pregnancy; terminal stages of incurable chronic diseases; injuries sustained while under the influence of alcohol or drugs; and military injuries.

The study protocol received approval from the local ethics committee of the FRCC ICMR (protocol No. 2/23/4 dated May 30, 2023). Written informed consent was obtained from all patients or their legal representatives.

### 2.2 Clinical assessment

Upon admission, the severity of patients’ condition was assessed using the Sequential Organ Failure Assessment (SOFA) and Glasgow Coma Scale (GCS). A SOFA score of 3 or higher for any organ was defined as organ failure. For GCS, a score of 3 to 8 indicated severe TBI, while a score of 9 to 12 indicated moderate TBI (31). Patients were categorized into moderate and severe TBI groups based on their initial Glasgow Coma Scale (GCS) scores at the acute stage. While we recognize that Post-Traumatic Amnesia (PTA) and Loss of Consciousness (LOC) are standard criteria for TBI classification, these metrics were not uniformly available in the retrospective records from all referring acute care facilities. Consequently, GCS remained the primary metric for severity categorization in this cohort, and all included patients met the criteria for significant neurotrauma requiring intensive care or specialized rehabilitation (29).

### 2.3 Quantitative assessment of TREC and KREC

TREC and KREC levels were determined in whole venous blood samples using multiplex real-time polymerase chain reaction (PCR-RT), as described previously (24, 27). DNA was extracted from 200 μL of blood via isopropanol precipitation. The PCR-RT reaction mixture (25 μL total volume) contained 200 ng of genomic DNA. It also included specific primers and probes for the 8REC-Jα T-cell receptor (TREC) fragment, the kappa deletion junction (KREC) region, and the human albumin gene (serving as the reference gene). Amplification was performed on a CFX96 instrument (Bio-Rad, United States) using the following program: 95 °C for 7 min, followed by 45 cycles of 93 °C for 30 s and 59 °C for 1 min. For absolute quantitative assessment, standard calibration curves were generated from serial dilutions of plasmid constructs containing target sequences for TREC, KREC, and the albumin gene. The number of TREC and KREC copies per 100,000 nucleated cells was calculated using the following formula: Number of copies = [average number of TREC (KREC) copies/(average number of albumin gene copies/2)] × 100,000.

### 2.4 Data collection and observation design

TREC/KREC measurements were collected for all patients. For 22 patients, only a single measurement was available upon admission due to technical reasons. For the remaining 29 patients, two or more measurements were taken over time. The study design primarily involved comparing TREC/KREC levels at admission and before discharge. However, additional measurements were obtained when a significant change in the patient’s clinical condition (either improvement or deterioration) occurred. Signs of deterioration included, in particular: the development of infectious complications, such as the appearance of fever, clinical and laboratory signs of sepsis or septic shock, the development of nosocomial pneumonia confirmed by X-ray; increased organ dysfunction on the SOFA scale; change in neurological status: sudden decrease in level of consciousness, development of status epilepticus. Key positive events included: successful discontinuation of mechanical ventilation (decannulation), restoration of consciousness, significant regression of neurological deficit, preparation for discharge.

Among patients with repeated measurements, the median number of measurements was 3 (IQR 2–4), covering a typical observation window of 30 days (IQR 16–69) since hospital admission.

### 2.5 Statistical analysis

Statistical analyses were conducted using Python (v3.13) with the statsmodels, pandas, and scikit-learn libraries. For the multivariate analysis, three patients were excluded due to missing clinical scores, resulting in a final analytical sample of 48 patients (112 total observations). To ensure statistical validity, TREC and KREC levels were log_10_(*x* + 1) transformed to achieve normality and stabilize variance.

#### 2.5.1 Overview of analytical strategy

To ensure rigorous model selection and validation, our statistical workflow followed a structured four-step approach: (i) Base Model Selection: Determining the optimal temporal scale (time from injury vs. hospitalization) and identifying key demographic covariates. (ii) Dimensionality Reduction: Addressing multicollinearity among clinical predictors using Principal Component Analysis (PCA). (iii) Model Optimization: Selecting the best combination of predictors and testing for interactions using hierarchical regression based on the corrected for small sample sizes Akaike Information Criterion (AICc). (iv) Robustness Validation: Verifying the stability of the final models using nonparametric cluster bootstrapping.

#### 2.5.2 Linear mixed-effects model (LMM) specification

To account for the non-independence of repeated measurements, we employed Linear Mixed-Effects Models (LMMs). In all models, patient ID was specified as a random intercept. The primary dependent variables (outcomes) were the log-transformed levels of TREC and KREC. Fixed effects candidates included demographic factors (mean-centered age, sex), temporal scales, and clinical indicators of severity and inflammation.

#### 2.5.3 Step 1: temporal scale and base model selection

We first sought to identify the optimal temporal scale for modeling immune restoration. We explicitly compared two scales: biological time (days since injury, calculated as days since hospitalization plus injury-to-hospitalization lag) and clinical time (days since hospitalization). “Lag” is a fixed number of days for each patient representing the time between injury and hospitalization. We also tested for non-linear effects of time by comparing linear models against those with a quadratic (squared) term.

Based on AICc and Log-Likelihood comparisons, clinical time was identified as the superior fit for TREC, while biological time was preferable for KREC. To determine if the initial injury timing influenced later recovery, we tested a combined model incorporating clinical time alongside a fixed covariate for the “injury-to-hospitalization lag.” As this lag term was not significant (*p* > 0.25, Supplementary Table S3), clinical time was retained as the primary temporal predictor for the final multivariate analysis.

Reference norms from healthy populations were used as external benchmarks to contextualize the degree of immune depletion. While these norms were not used as mathematical offsets within the LMM (to maintain focus on intra-cohort dynamics), they provided the basis for the descriptive analysis of baseline immune suppression.

#### 2.5.4 Step 2: dimensionality reduction (composite index)

To address significant multicollinearity among clinical markers—Sequential Organ Failure Assessment (SOFA) score, Neutrophil-Lymphocyte Ratio (NLR), and C-reactive protein (CRP)—we utilized Principal Component Analysis (PCA) as an *a priori* data reduction technique. The first principal component (PC1), which explained 66.4% of the total variance and showed consistent positive loadings for all three markers, was retained as a composite “severity/inflammation index.”

#### 2.5.5 Step 3: hierarchical regression and interaction testing

Final variable selection was performed using a hierarchical regression approach. We compared nested models of increasing complexity—starting from a base demographic/temporal model and sequentially adding the severity/inflammation index, absolute lymphocyte counts, and potential interaction terms—using the corrected Akaike Information Criterion (AICc) to identify the most parsimonious model for TREC and KREC independently. Following selection, we explicitly tested for interactions between time and systemic inflammation to determine if recovery rates were moderated by the severity of the inflammatory response.

#### 2.5.6 Step 4: robustness and validation

The stability of the final multivariate models was validated using a nonparametric cluster bootstrap procedure (1,000 iterations). Resampling was performed at the patient level (blocks) to preserve longitudinal correlations. Model quality was further assessed through parameter bias analysis and investigation of the parameter correlation matrix. A *p*-value of < 0.05 was considered statistically significant.

## 3 Results

### 3.1 Sample characteristics

**Figure 1:**
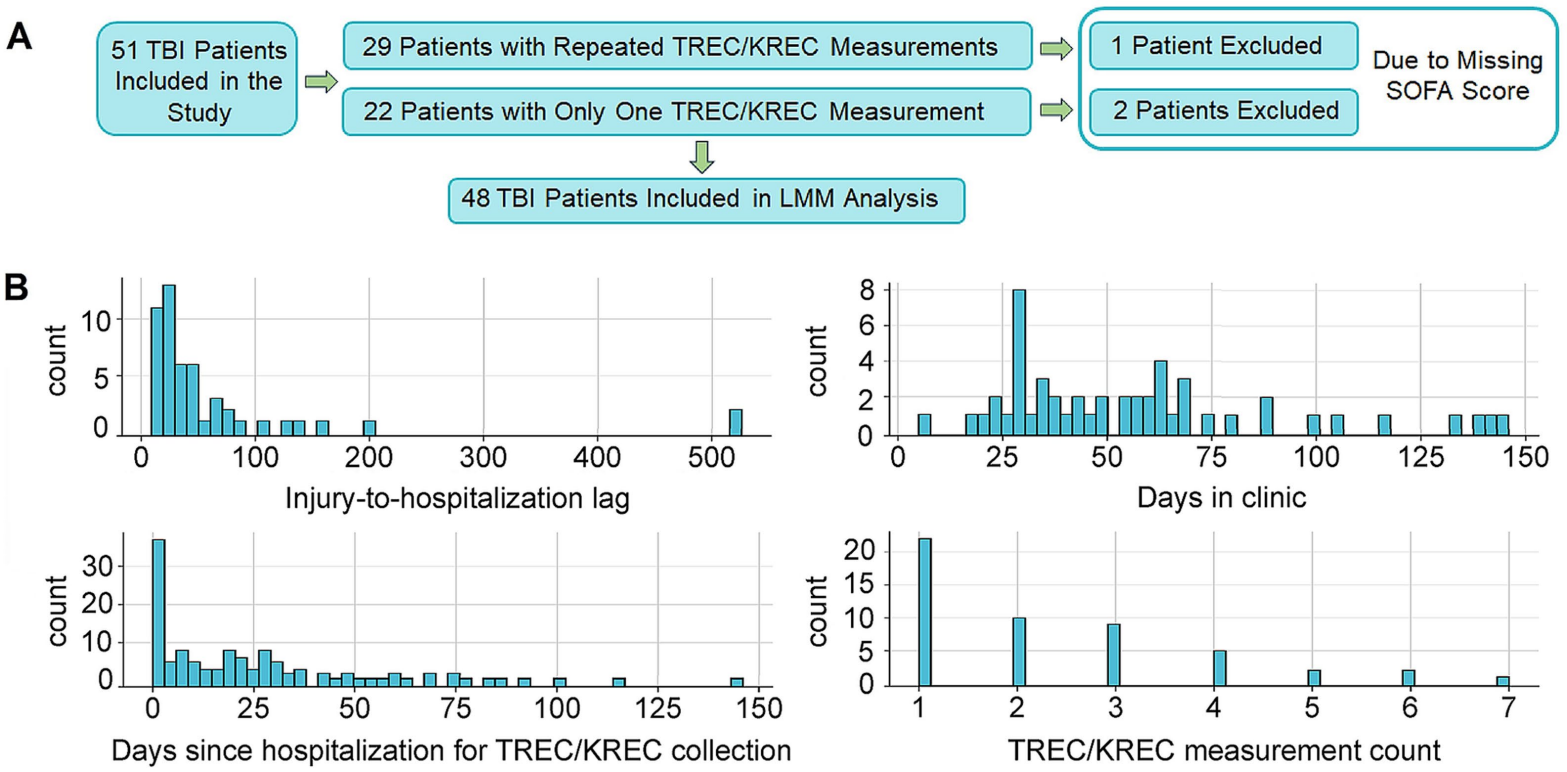
Study design and distribution of patient data. **(A)** Flowchart illustrating the patient selection process. Of the total cohort (*n* = 51), a subgroup of 29 patients with repeated measurements was used for longitudinal analysis. For the construction of the linear mixed model (LMM), a further subgroup of 48 patients was included after excluding three patients with missing observations. **(B)** Distribution of key temporal and measurement parameters across the patient cohort. Histograms show the frequency of patients by (i) fixed number of days for each patient representing the time between injury and hospitalization (lag) (ii) total days spent in the clinic, (iii) days since hospitalization for TREC/KREC collection, and (iv) the total number of TREC/KREC measurements performed per patient.

The study cohort comprised 51 patients with moderate-to-severe TBI. Figure 1 provides an overview of the study design, detailing patient selection for subgroup and LMM analyses (Figure 1A), and the distribution of clinical timelines and TREC/KREC measurement frequencies (Figure 1B). Demographic, clinical, and injury-related characteristics are summarized in Table 1. The median duration of the rehabilitation stay was 31.5 days (IQR 24–40). Upon admission, the median SOFA and GCS scores were 3 (IQR 1–4) and 10 (IQR 8–14), respectively.

**Table 1:** Clinical presentation of TBI patients.

Characteristics	*N* (%) or Me (IQR)
Demographic characteristics	
Male	41 (80.39)
Female	10 (19.60)
Age	40 (31–47)
Injury mechanism	
Falls	21 (41.18)
Transport accidents	18 (35.29)
Industrial injuries	6 (11.76)
Assault	3 (5.88)
Undetermined	3 (5.88)
Types of TBI	
Closed brain injury	36 (70.59)
Penetrating brain injury	15 (29.41)
TBI Characteristics	
Any extracranial injury	38 (74.5)
Polytrauma (≥2 body regions injured)	28 (54.9)
Thoracic injuries	19 (37.3)
Rib fractures	15 (29.4)
Pulmonary contusion	12 (23.5)
Hemothorax/pneumothorax	11 (21.6)
Abdominal and pelvic injuries	8 (15.7)
Pelvic fracture	7 (13.7)
Solid organ injury	1 (2.0)
Extremity injuries	19 (37.3)
Upper extremity fracture	11 (21.6)
Lower extremity fracture	7 (13.7)
Spinal injuries	10 (19.6)
Cervical spine fracture	2 (3.9)
Thoracic spine fracture	4 (7.8)
Lumbar spine fracture	5 (9.8)
Transverse/spinous process fracture	7 (13.7)
Facial injuries	16 (31.4)
Physiological insult	2 (3.9)
Hemorrhagic shock	2 (3.9)
Surgical interventions after TBI	
Surgical interventions	40 (78.43)
Top 5 Nervous/Mental Complications	
Mental disorder due to brain damage and dysfunction and to physical disease	35 (68.63)
Flaccid neuropathic bladder	29 (56.86)
Dysphagia	27 (52.94)
Paraplegia and tetraplegia	18 (35.29)
Cerebral edema	14 (27.45)
Top 5 Infectious Complications	
Nosocomial pneumonia	37 (72.55)
Urinary tract infection	10 (19.61)
Bacterial meningitis	5 (9.80)
Sepsis	3 (5.88)
Intracranial abscess and granuloma	2 (3.92)
Other Top 5 Complications	
Iron deficiency anemia	22 (43.14)
Decubitus ulcer and pressure area	19 (37.25)
Protein-energy malnutrition	14 (27.45)
Gastric ulcer/ Duodenal ulcer/ Peptic ulcer	12 (23.53)
Venous embolism and thrombosis	7 (13.73)
Devices	
Tracheostomy	40 (78.43)
CVC	19 (37.25)
Urethral catheter	11 (21.57)
Cystostomy	6 (11.76)
Nasogastric tube	4 (7.84)
Patients without devices	11 (21.57)
Critical care admission and outcome	
ICU admission	20 (39.22)
Artificial ventilation	16 (31.37)
Deceased	3 (5.88)
Clinical scales score at admission	
Sequential Organ Failure Assessment (SOFA) score	3 (1–4)
Glasgow Coma Scale (GCS) Score	10 (8–14)
Clinical scales score at discharge	
SOFA	2 (0–3)
GCS	12 (9–15)

Concomitant extracranial injuries were highly prevalent, contributing to the overall physiological burden; only 25.5% of patients presented with isolated TBI. The remaining 74.5% sustained at least one extracranial injury, and 54.9% met the criteria for polytrauma. Thoracic trauma and extremity injuries were each present in 37.3% of the cohort. Spinal injuries were documented in 19.6% of patients, while abdominal and pelvic injuries were observed in 15.7%. Facial fractures were present in 31.4% of cases. This high prevalence of extracranial trauma—particularly thoracic and orthopedic injuries—likely amplified the systemic inflammatory response and secondary infectious risk.

Consistent with the severity of injury and prolonged immobilization, the cohort exhibited a substantial pathogenic burden. Nosocomial pneumonia was the predominant infectious complication (72.6%), and three patients (5.9%) developed fatal sepsis. This high rate of infection corresponded to extensive exposure to invasive medical devices: 78.4% of patients underwent tracheostomy, and central venous catheters were utilized in 37.3%. Only 21.6% of patients remained free of invasive devices during their admission.

Secondary morbidity was nearly universal, with 68.6% of patients diagnosed with mental disorders secondary to brain injury. Systemic complications reflective of chronic critical illness, such as iron deficiency anemia (43.1%) and protein-energy malnutrition (27.5%), were also prevalent. Collectively, these characteristics describe a cohort with high metabolic demands, significant requirement for life-sustaining interventions, and pronounced susceptibility to nosocomial complications.

### 3.2 TREC and KREC levels upon patient admission

**Figure 2:**
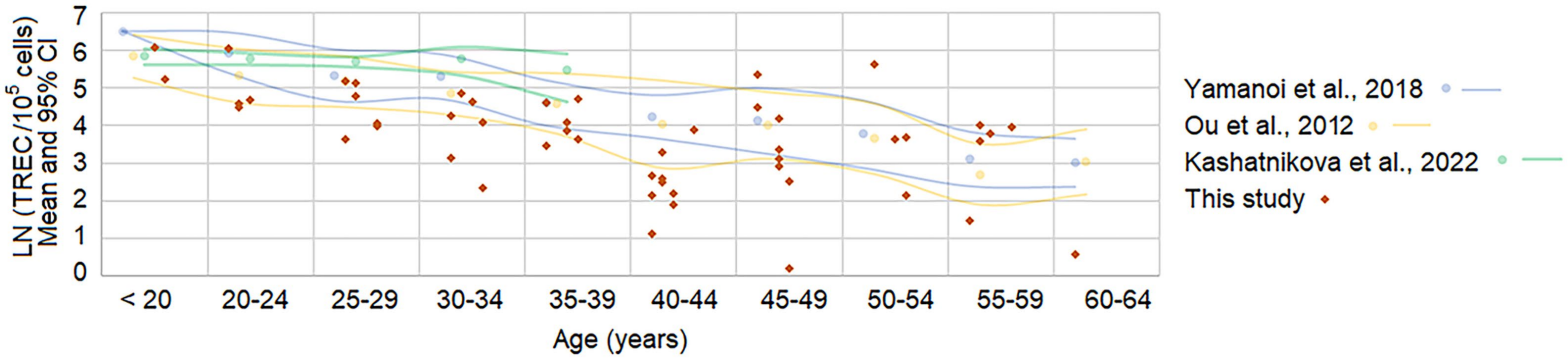
TREC levels in patients with TBI at admission compared to population reference ranges. Individual TREC levels from 51 TBI patients are shown as red diamonds. Data from three different control populations are represented by colored dots (mean values) and smoothed lines (95% confidence intervals). The *Y*-axis displays natural logarithmic TREC values (copies/10^5^ cells), and the *X*-axis shows patient age in 5-year intervals.

As TREC levels have not been previously studied in patients with moderate-to-severe TBI, we first compared the baseline TREC levels in our cohort with published data from healthy populations (Figure 2). For a meaningful comparison, we selected three studies from the literature that presented data in 5-year age intervals and used compatible units (TREC copies per 10^5^ cells or dCt values) (24, 32, 33). Other studies were excluded from the main analysis as their converted values were an order of magnitude higher, precluding direct comparison (34, 35) (Supplementary Methods and Supplementary Table S1). The majority of our patients, across all age brackets, had TREC levels at or below the lower limit of the age-specific normal range.

### 3.3 Dynamics of TREC and KREC levels in patients with TBI

Longitudinal dynamics, analyzed in 29 patients with repeated measurements, showed substantial variability. For TREC, 18 patients exhibited an increasing trend over time, while 11 showed a decreasing trend. Notably, the three patients with decreasing TREC dynamics were those who developed fatal sepsis. KREC dynamics were similarly variable: 14 patients showed an increasing trend and 15 had a decreasing trend.

**Figure 3:**
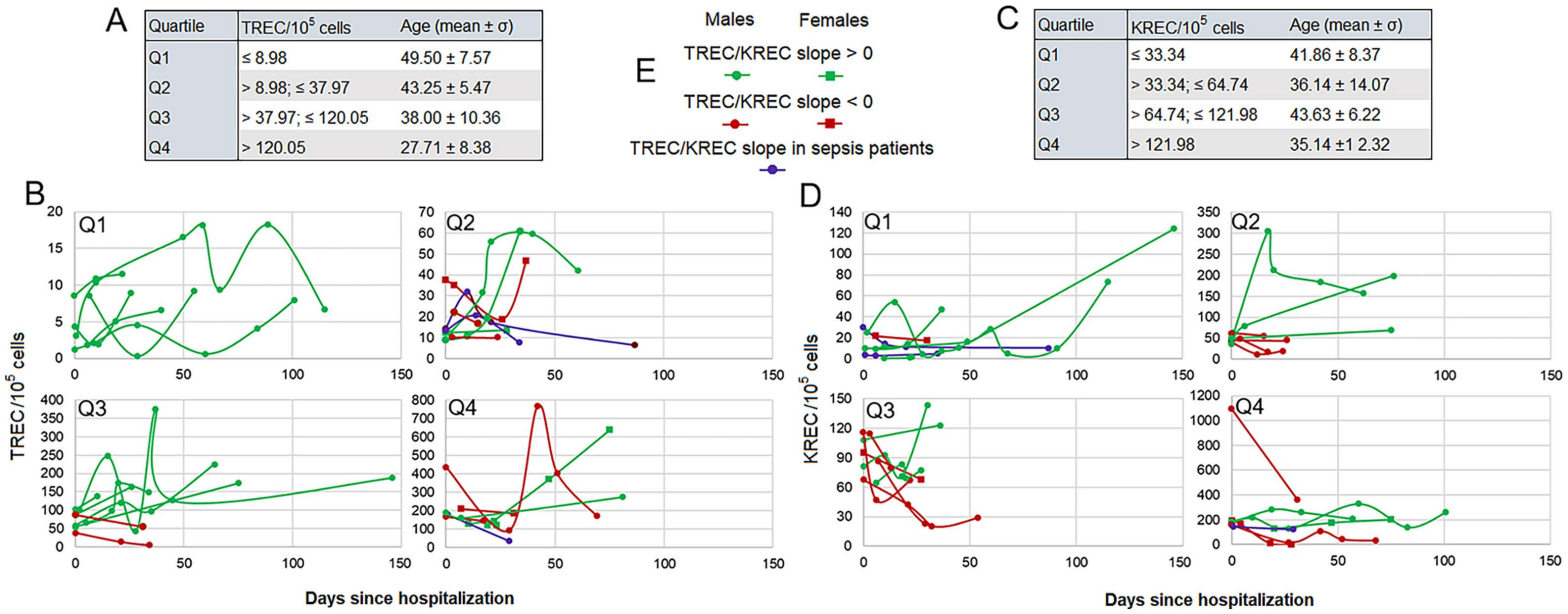
Individual dynamics of TREC and KREC levels in patients (*n* = 29) stratified by baseline quartiles. **(A)** Characteristics of TREC quartiles, including value ranges and mean patient age. **(B)** Individual TREC dynamics over time within each quartile. **(C,D)** Corresponding quartile characteristics and individual dynamics for KREC. In panels **(B,D)**, each line represents a single patient. **(E)** Legend for the patient dynamic plots.

Stratification into quartiles based on baseline levels (Figures 3A,C) revealed a significant difference in age across TREC quartiles (ANOVA, *p* = 0.0011), a pattern not observed for KREC (*p* = 0.41). A hallmark feature across all quartiles was the presence of highly fluctuating, non-monotonic changes, with some individuals experiencing multi-fold variations over the course of weeks (Figures 3B,D,E).

### 3.4 Base model selection and characteristics (step 1)

Baseline Linear Mixed-Effects Models (LMM) were constructed to establish the fundamental drivers of TREC and KREC dynamics.

#### 3.4.1 Comparison of temporal scales and assessment of non-linearity

We sought to determine whether immune recovery was more closely associated with biological time (days since injury) or clinical time (days since hospitalization). As shown in Supplementary Table S2, the temporal dynamics differed by marker. For TREC, modeling biological time (days since injury) revealed a significant concave (quadratic) trajectory (*p* = 0.044), suggesting an initial rapid increase followed by a plateau. Conversely, for KREC, clinical time (days since hospitalization) provided a superior fit (Delta AICc > 12) and also exhibited a significant quadratic pattern (*p* < 0.001), contrasting with the linear trajectory observed on the biological timescale.

A combined model incorporating both clinical time and the injury-to-hospitalization lag yielded a marginally improved fit, though the lag term itself was not a significant predictor (Supplementary Table S3). This confirms that time spent within the controlled rehabilitation environment is the primary driver of the observed recovery patterns.

#### 3.4.2 Base effects and variance partitioning

The base LMMs established age and sex as the primary determinants of baseline TREC levels, with males and older patients exhibiting lower counts. Days since hospitalization showed a positive trend but did not reach statistical significance at this stage (*p* = 0.081) (Supplementary Table S4). In contrast, the KREC base model (Supplementary Table S4) showed no significant associations with age, sex, or days since hospitalization.

Variance partitioning revealed an Intraclass Correlation Coefficient (ICC) of 64.2% for TREC and 66.0% for KREC. This indicates that while inter-individual differences are the dominant factor, approximately 34–36% of the total variance is driven by intra-individual changes over time, justifying the search for longitudinal clinical predictors.

Furthermore, we investigated whether the effect of age on TREC differed between sexes by including an interaction term. An interaction term (age × sex) was non-significant (*p* = 0.764), justifying the use of additive models (Supplementary Table S5).

### 3.5 Collinearity testing and composite index creation (step 2)

Assessment of primary clinical predictors (SOFA, NLR, and CRP) revealed strong inter-correlations (*r* = 0.48 to 0.68). To mitigate multicollinearity, we used Principal Component Analysis (PCA) to create a single composite “severity/inflammation index” (Supplementary Figure S1). The loadings were all positive and similar in magnitude, confirming PC1 as a representative proxy for systemic inflammatory and organ-system stress.

### 3.6 Hierarchical model selection (step 3)

Hierarchical model comparison (Supplementary Table S6) demonstrated that TREC dynamics are heavily moderated by systemic factors, as the inclusion of the severity/inflammation index significantly improved model fit (Delta AICc = −24.01). In contrast, for KREC, the base demographic model remained the most parsimonious (AICc = 149.71), as clinical indices provided no additional explanatory power (Delta AICc > 1.1). Notably, absolute lymphocyte count was not a top predictor for either marker, suggesting that TREC/KREC density provides unique biological information beyond total cell quantity.

#### 3.6.1 Drivers of TREC recovery

**Figure 4:**
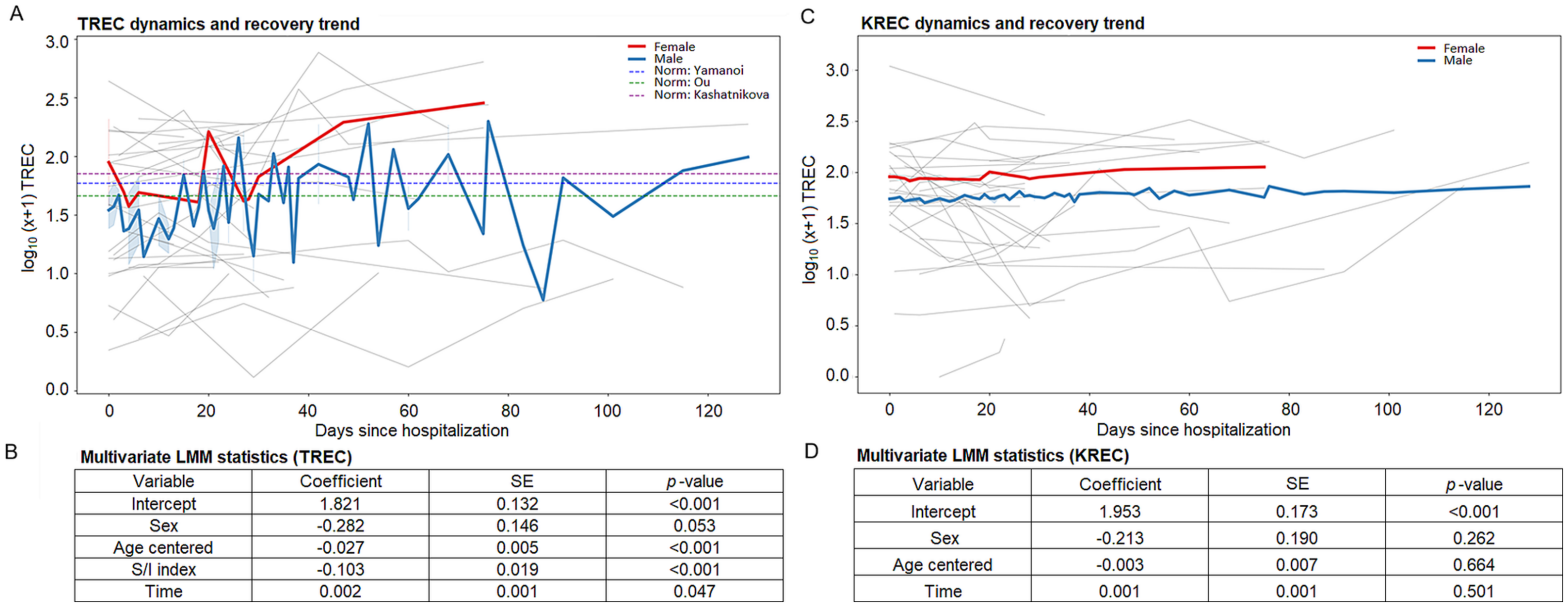
Modeled dynamics of TREC and KREC during hospitalization. **(A,C)** Longitudinal trajectories of TREC and KREC. Thin light-gray lines represent individual raw patient data, illustrating the high inter-individual variability. Thick colored lines represent the predicted mean recovery trends for males (blue) and females (red) derived from the linear mixed model. The shaded areas (shadows) represent the 95% confidence intervals of these predictions. **(A)** Horizontal dashed lines represent cohort-wide mean reference norms from three independent studies: Kashatnikova et al. (24), Ou et al. (32), and Yamanoi et al. (33). **(B,D)** Summary tables providing the exact coefficients, standard errors, and *p*-values for the final multivariate models. S/I index, severity/inflammation index; SE, standard error; Time, days since hospitalization.

The final TREC multivariate model (Supplementary Table S7 and Figure 4) identified the severity/inflammation index as a powerful negative predictor (Coef: −0.1025, *p* < 0.001). Crucially, adding this index reduced the within-patient (residual) variance by 26.6% (from 0.0719 to 0.0528), increasing the ICC to 69.8%.

Adjusting for inflammation also unmasked the effect of time: days since hospitalization became a significant positive predictor (Coef: 0.002, *p* = 0.047), indicating that T-cell homeostasis steadily recovers as the acute physiological stress resolves.

Beyond main effects, we explored potential interactions between days since hospitalization and severity/inflammation index, however the results were non-significant (Supplementary Table S8).

Visual inspection of predicted recovery trajectories (Supplementary Figure S2) suggests that while patients with lower systemic inflammation exhibit a robust recovery of TREC levels during rehabilitation, higher baseline inflammation may suppress the rate of naive T-cell homeostatic restoration.

#### 3.6.2 Predictors of KREC dynamics

The KREC model confirmed that B-cell dynamics were not robustly explained by the examined clinical parameters (*p* > 0.25 for all predictors) (Supplementary Table S7 and Figure 4). Using hierarchical model comparison, the base demographic model remained the most parsimonious (AICc = 149.71), with the addition of clinical indices providing no significant improvement in fit (Delta AICc > 1.1). This suggests that while KREC levels exhibit significant inter-individual variability, their trajectories remain independent of the severity of the initial injury or the degree of systemic inflammation.

### 3.7 Nonparametric bootstrap stability (step 4)

Model estimates obtained via the nonparametric cluster bootstrap (1,000 iterations) were stable, with 95% bootstrap confidence intervals for age, sex, and the severity/inflammation index excluding zero (Supplementary Table S9). Bias analysis confirmed high model quality with minimal parameter bias across all predictors. A comparison of asymptotic and bootstrap confidence interval (CI) widths revealed that bootstrap CIs were generally comparable to the original estimates, reflecting high model stability, as demonstrated for the severity/inflammation index (Supplementary Figure S3), and the correlation matrix (Supplementary Table S10) showed that the model effectively partitioned inter-patient variance.

### 3.8 Synchronous immune, clinical, and neurological recovery

**Figure 5:**
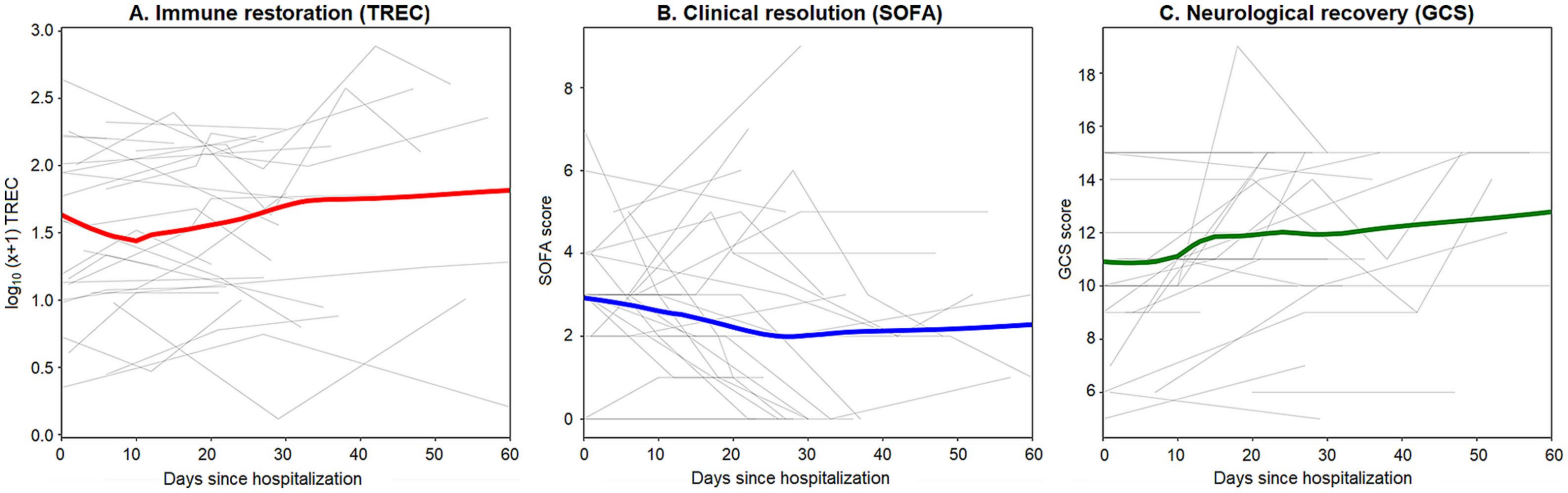
Comparative trajectories of immune restoration, clinical stabilization and neurological improvement. **(A)** Longitudinal trajectories of TREC levels. Thin gray lines represent individual patient kinetics (*n* = 29 with repeated measures); the thick red line represents the cohort-wide smoothed trend (LOWESS). **(B)** Longitudinal trajectories of Sequential Organ Failure Assessment (SOFA) scores for the same cohort. The thick blue line indicates the steady resolution of organ dysfunction over the hospitalization period. **(C)** Longitudinal trajectories of Glasgow Coma Scale (GCS) scores for the same cohort. The thick green line shows GCS increases (improvement in consciousness/function).

To contextualize the biological significance of TREC restoration, we compared longitudinal immune trajectories against clinical and functional recovery markers during the first 60 days of rehabilitation. As illustrated in Figure 5, the cohort exhibited a synchronous recovery pattern characterized by a gradual rise in TREC levels occurring in tandem with the resolution of organ dysfunction (declining SOFA scores) and the improvement of neurological status (rising GCS scores). While individual trajectories remained stochastic, these aggregate trends highlight that the stabilization of naive T-cell homeostasis is a fundamental biological component of the broader recovery process in moderate-to-severe TBI.

## 4 Discussion

In this study, we investigated the levels and longitudinal dynamics of TREC and KREC in patients undergoing rehabilitation after TBI, and characterized their associations with immune status and systemic recovery. Our principal findings demonstrate that most TBI patients exhibit significantly depleted TREC levels compared to age-matched healthy norms, reflecting a compromised naive T-cell homeostatic capacity during the subacute phase. While longitudinal trajectories were characterized by substantial inter-individual variability and non-monotonic fluctuations, multivariate analysis identified age, sex, and systemic inflammation as the primary drivers of TREC restoration. TREC-level recovery occurred alongside neurological improvement (increasing GCS) and resolution of organ dysfunction (decreasing SOFA). In contrast, KREC levels were remarkably independent of the parameters considered.

The observation of low baseline TREC levels highlights the necessity of age-matched reference intervals; a value normal for an older adult may signify profound immunodepletion in a younger patient (17, 36). This state of impaired naive T-cell homeostasis aligns with the concept of “chronic critical illness” (CCI) (37), where patients surviving the acute phase of severe TBI experience persistent inflammation, immunosuppression, and catabolism (38–41). Biologically, TREC levels are determined by the interplay between thymic output and homeostatic proliferation. While thymic contribution naturally diminishes with age—falling from 20% at age 25 to just 5% after age 55 (42)—the peripheral pool is maintained by the division of existing cells. This homeostatic proliferation “dilutes” TRECs within the population (43), and cells undergoing extensive division are often less capable of forming memory or skewing subset polarization (44–46). Thus, the TREC depletion observed in our cohort likely serves as a molecular signature of the exhausted regenerative capacity associated with CCI.

Interpreting the rapid, multi-fold fluctuations in TREC levels observed over weeks requires looking beyond thymic output alone (47, 48). These dynamics likely reflect the immune system’s reactive state, driven by peripheral factors such as the diluting effect of proliferation, rapid lymphocyte trafficking between blood and lymphoid tissues, and shifts in fluid status (49–51). A transient decrease in TRECs is not inherently deleterious; it may signify a robust proliferative response or increased immune surveillance (52, 53). However, when viewed alongside the overall clinical picture, a persistent downward trend signifies a failure of homeostatic resilience and a depleted regenerative reserve (41, 54).

Our analysis reveals a striking synchrony between the restoration of naive T-cell homeostasis and clinical recovery, as evidenced by the parallel improvements in neurological status (GCS) and the resolution of multi-organ dysfunction (SOFA). This alignment suggests that TREC levels may serve as an objective biological barometer for systemic recovery (55, 56). While clinical scores like the GCS can be confounded by sedation or observer variability (57), the gradual rise in TRECs provides a direct readout of the transition from a catabolic, injury-driven state to an anabolic, recovery-permissive environment. In this context, “clinical time” serves as a proxy for physiological stabilization. Intensive nutritional support and the correction of iron and protein-energy deficits likely mitigate initial stress-induced perturbations, facilitating T-cell maintenance through metabolic repletion even in the face of ongoing nosocomial challenges (58, 59).

Notably, TREC analysis provided granular insights that traditional markers failed to capture. For instance, GCS score upon admission was not a significant predictor of TREC levels, likely because admission GCS reflects acute neurological insult (6), whereas TREC levels represent the cumulative, evolving state of systemic immune health. Similarly, total lymphocyte count was not associated with TREC levels. Total counts are crude measures that amalgamate diverse functional subsets; a patient may possess a numerically normal lymphocyte count that is functionally deficient, characterized by a depleted pool of new, “high-quality” naive T-cells (60).

The value of TREC analysis lies in its ability to move beyond simple correlation to reveal an underlying biological phenotype. While chronological age is fixed, TREC levels offer a quantitative measure of “immune age,” potentially explaining why patients of the same age experience vastly different trajectories. By consolidating the impact of inflammation, metabolic stress, and organ dysfunction into a single readout, TREC monitoring shifts the paradigm toward “immuno-rehabilitation.” This aligns with the recently proposed NIH-NINDS TBI Classification and Nomenclature Initiative (61), which advocates for a multidimensional “Pillar” approach to capture injury heterogeneity. Our findings of synchronous immune and clinical recovery support the call for dynamic, repeated assessments to determine how biological pillars evolve during the sub-acute and chronic stages.

The asynchronous fluctuations observed in KREC levels, which did not correlate with TREC dynamics or systemic inflammation, suggest that B-cell and T-cell homeostasis are governed by distinct drivers in the post-TBI environment (62–64). This differential impact warrants separate investigation to understand the specific vulnerabilities of the humoral immune arm following neurotrauma.

From a clinical perspective, TREC quantification offers a tool for real-time risk stratification. A “flexible” immune system—where TRECs recover quickly as inflammation subsides—suggests favorable resilience. Conversely, a “rigid” or persistently low TREC profile despite clinical improvement may indicate that T-cells are sequestered in tissues (65, 66), anergic (67), or depleted by apoptosis (68), rendering the patient vulnerable to secondary infections.

### 4.1 Limitations and future directions

This study is limited by its small sample size, single-center design, and clinical heterogeneity regarding injury mechanisms. The variable timing of enrollment (16–69 days post-injury) was addressed by comparing biological and clinical time scales, but the long-term influence of the initial injury complex remains to be fully elucidated. Future research should co-measure markers of cellular proliferation (e.g., Ki-67) and trafficking (e.g., CCR7 and CD62L) to deconstruct the complex signals of TREC fluctuations in critical illness (52, 69). Such a multi-marker approach would clarify whether rapid changes are driven by homeostatic expansion or lymphocyte redistribution.

While the current study focuses on the determinants of naive T- and B-cell restoration, the potential of TREC and KREC as prognostic biomarkers (independent predictors of clinical outcome) remains a critical area for future investigation. Our findings regarding the influence of age and systemic inflammation provide the necessary framework for interpreting such prognostic models in larger, multicenter cohorts.

## 5 Conclusion

In conclusion, TREC levels in TBI patients are closely linked to age and the resolution of systemic inflammation. These dynamics reflect the stability and reactivity of naive T-cell homeostasis rather than thymic output alone, positioning TREC as a promising biological barometer for recovery in neurocritical care and other forms of chronic critical illness.

## Data Availability

The raw data supporting the conclusions of this article will be made available by the authors, without undue reservation.
