# Supplementary figures and images for: TREC dynamics as a biomarker of naive T-cell homeostasis in traumatic brain injury: a longitudinal analysis
Source: Front Med (Lausanne). 2026 Mar 12;13:1775886. doi: 10.3389/fmed.2026.1775886 (PMC13018110; doi:10.3389/fmed.2026.1775886)

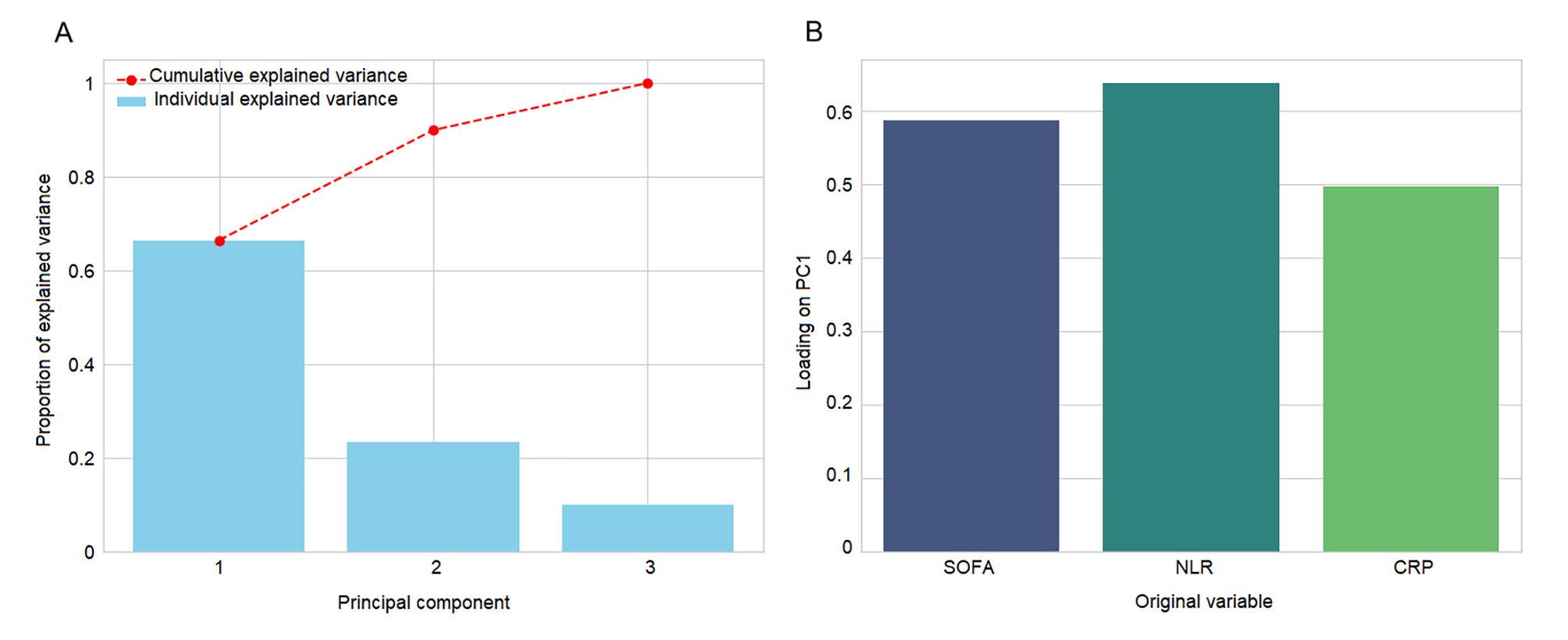

Supplement: Supplementary file 2 [file Image_1.tif]

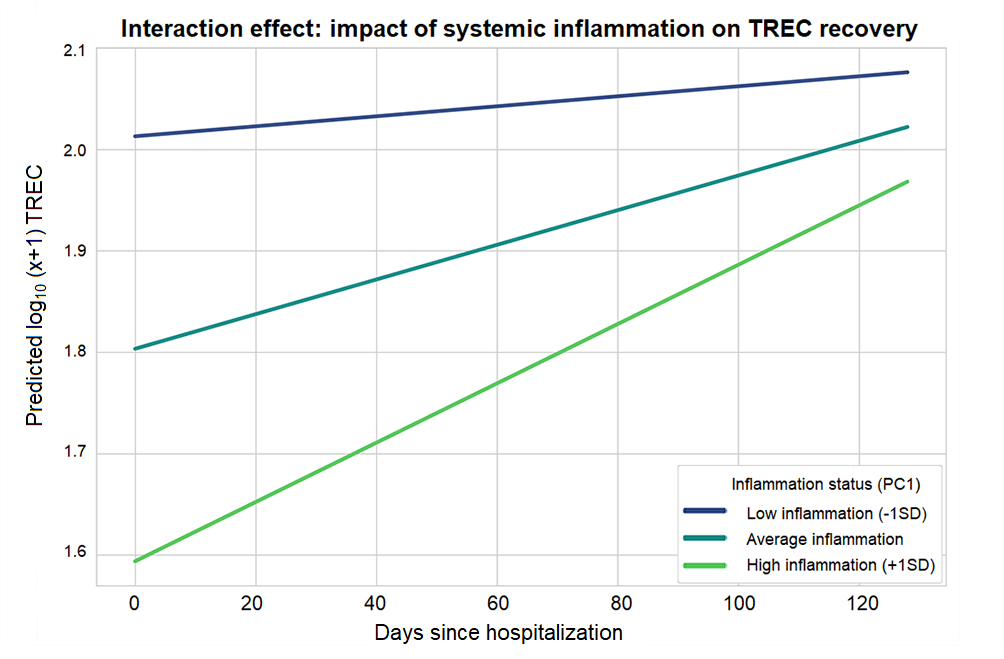

Supplement: Supplementary file 3 [file Image_2.tif]

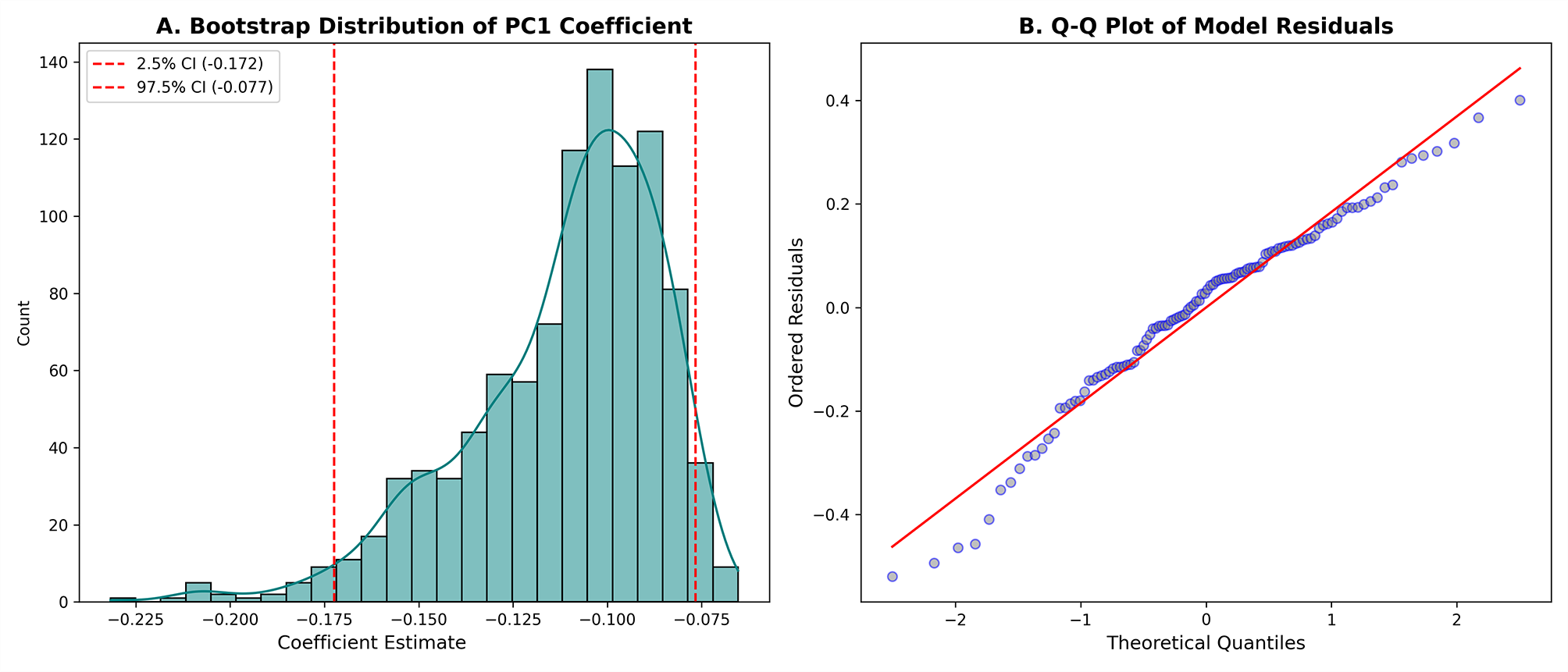

Supplement: Supplementary file 4 [file Image_3.tiff]
