# Supplementary material for: TREC dynamics as a biomarker of naive T-cell homeostasis in traumatic brain injury: a longitudinal analysis
Source: Front Med (Lausanne). 2026 Mar 12;13:1775886. doi: 10.3389/fmed.2026.1775886 (PMC13018110; doi:10.3389/fmed.2026.1775886)
Supplement: Supplementary file 5 [file Table_1.docx]

**Supplementary Material**

**Supplementary Methods:** Standardization of TREC Data from Literature

To compare our results with published data, TREC measurements from several studies were converted to a standardized unit of "copies per 10^5 cells". The conversion algorithms differed based on the original units reported in each study.

1. Conversion from Log(copies/µg DNA) (as in Cho et al., 2014) (**1**)

This conversion is based on the assumption that a single human diploid leukocyte contains approximately 6.6 pg of DNA (https://bionumbers.hms.harvard.edu/bionumber.aspx?id=111206).

Step 1: Linearization. Convert logarithmic copies per 1 µg DNA (L) to a linear scale: TREC copies per 1 µg DNA = 10^L.

Step 2: Normalization. Calculate the equivalent amount of DNA in 10^5 cells (0.66 µg) and apply the conversion factor: TREC copies per 10^5 cells = (TREC copies per µg DNA) × 0.66.

2. Conversion from Log(copies/mL blood) (as in Lorenzi et al., 2008) (**2**)

This conversion assumes an average lymphocyte concentration of 2 x 10^6 cells/mL in adult blood (https://www.pacehospital.com/lymphocyte-count-normal-range).

Step 1: Linearization. Convert log copies per mL to a linear scale: TREC/mL = 10^(LogTREC/mL).

Step 2: Normalization. Normalize the count based on the assumed lymphocyte concentration: TRECs per 10^5 cells = (TREC/mL / 2,000,000) × 100,000 = TREC/mL / 20.

3. Conversion from dCt values (as in Ou et al., 2012 & Yamanoi et al., 2018) (**3, 4**)

This relative quantification method uses the TATA-binding protein (TBP) gene as an endogenous reference.

Step 1: Calculate TREC/TBP ratio. The ratio is calculated from the difference in threshold cycles (dCt = Ct_TBP - Ct_TREC) using the formula 2^dCt.

Step 2: Normalize to 10^5 cells.* Since one diploid cell contains two copies of the TBP gene, the final formula is: TRECs per 10^5 cells = 2 × 10^5 × 2^dCt.

**Supplementary Table S1.** Raw and recalculated TREC data from reference studies

| Cho et al., 2014 (**1**) | | | |
| --- | --- | --- | --- |
| Group (age) | *n* (# of persons in the group) | Mean  (Log [sjTREC per µg DNA]) | Mean TREC (95% CI) per 10^5^ cells (calculated) |
| 15–19 | 36 | 4.448 | 18516 |
| 20–24 | 21 | 4.352 | 14844 |
| 25–29 | 14 | 4.21 | 10704 |
| 30–34 | 18 | 4.181 | 10013 |
| 35–39 | 19 | 3.852 | 4694 |
| 40–44 | 13 | 3.836 | 4524 |
| 45–49 | 13 | 3.563 | 2413 |
| 50–54 | 16 | 3.487 | 2026 |
| 55–59 | 14 | 3.459 | 1899 |
| 60–65 | 8 | 3.023 | 696 |
| Lorenzi et al., 2008 (**2**) | | | |
| Group (age) | *n* (# of persons in the group) | Mean (LogTREC/ml) | Mean TREC per 10^5^ cells (calculated) |
| 15.1–20 | 4 | 4.64 | 2182.6 |
| 20.1–25 | 14 | 4.53 | 1694.2 |
| 25.1–30 | 9 | 4.55 | 1774.1 |
| 30.1–35 | 20 | 4.45 | 1409.2 |
| 35.1–40 | 16 | 4.12 | 659.1 |
| 40.1–45 | 9 | 4.16 | 722.7 |
| 45.1–50 | 16 | 4.05 | 561 |
| 50.1–55 | 23 | 3.84 | 345.9 |
| 55.1–60 | 15 | 3.68 | 239.3 |
| 60.1–65 | 10 | 3.48 | 151 |
| 65.1–70 | 4 | 3.84 | 345.9 |
| Ou et al., 2012 (**3**) | | | |
| Group (age) | *n* (# of persons in the group) | Mean±SE (dCtTBP-sjTREC) | Mean TREC (95% CI) per 10^5^ cells (calculated) |
| 15–19 | 13 | – 9.08±0.83 | 344.5 (193.8 – 612.3) |
| 20–24 | 15 | – 9.86±1.02 | 205.5 (100.3 – 421.0) |
| 25–29 | 19 | – 10.12±0.98 | 175.5 (89.2 – 345.3) |
| 30–34 | 19 | – 10.53±0.83 | 129.4 (72.8 – 229.9) |
| 35–39 | 27 | – 10.91±1.15 | 98.3 (44.0 – 220.0) |
| 40–44 | 34 | – 11.63±1.68 | 56.5 (17.7 – 180.7) |
| 45–49 | 26 | – 11.67±1.27 | 54.9 (22.7 – 132.6) |
| 50–54 | 23 | – 12.18±1.33 | 38.4 (15.1 – 97.4) |
| 55–59 | 8 | – 13.67±1.15 | 14.9 (6.7 – 33.3) |
| 60–64 | 6 | – 13.22±1.24 | 20.8 (8.8 – 49.3) |
| 65– | 11 | – 14.94±1.15 | 6.4 (2.9 – 14.3) |
| Yamanoi et al., 2018 (**4**) | | | |
| Group (age) | *n* (# of persons in the group) | Mean±SE (dCtTBP-sjTREC) | Mean TREC (95% CI) per 10^5^ cells (calculated) |
| 20-24 | 12 | – 8.92±0.79 | 375.9 (218.8 – 645.8) |
| 25-29 | 22 | – 9.57±0.99 | 207.3 (104.2 – 412.4) |
| 30-34 | 36 | – 9.62±0.86 | 200.1 (110.4 – 362.6) |
| 35-39 | 13 | – 10.59±0.88 | 99.5 (54.4 – 182.0) |
| 40-44 | 22 | – 11.04±0.82 | 69.6 (39.4 – 123.0) |
| 45-49 | 33 | – 11.18±1.22 | 62.8 (26.7 – 147.8) |
| 50-54 | 31 | – 11.73±1.3 | 43.5 (17.7 – 106.9) |
| 55-59 | 15 | – 12.59±1.04 | 22.6 (10.9 – 46.8) |
| 60–64 | 12 | – 12.73±0.93 | 20.4 (10.7 – 38.8) |
| > 65 | 6 | – 13.81±0.74 | 13.6 (8.1 – 22.9) |
| Kashatnikova et al., 2022 (**5**) | | | |
| Group (age) | n (# of persons in the group) | Mean TREC (± SE) per 10^5^ cells | Mean TREC (95% CI) per 10^5^ cells |
| <20 (18-19) | 50 | 346.31±36.18 | 346.3 (275.4 – 417.2) |
| 20-24 | 73 | 324.65±25.50 | 324.7 (274.7 – 374.6) |
| 25-29 | 15 | 300.39±83.75 | 300.4 (258.0 – 342.8) |
| 30-34 | 16 | 321.59±58.36 | 321.6 (207.2 – 435.98) |
| 35-39 | 15 | 236.02±67.55 | 236.02(103.6 – 368.4) |

**General Note on Supplementary Tables S2–S10:** All models were implemented as Linear Mixed-Effects Models (LMM) with a random intercept for patient ID. Age was mean-centered. Dependent variables were log_10_(x+1) transformed.

**Supplementary Table S2.** Comparison of model fit using days since injury and days since hospitalization as temporal scales for TREC and KREC, including a test for non-linearity for the days-since-injury scale

| # | Model | AICc | Log-Likelihood | Nonlinearity *p*-val |
| --- | --- | --- | --- | --- |
| TREC | | | | |
| 0 | Days since injury (linear) | 111.308 | -49.3813 | - |
| 1 | Days since injury (quadratic) | 109.4854 | -47.3574 | 0.0442 |
| 2 | Days since hospitalization | 109.162 | -48.3132 | - |
| 3 | Days since hospitalization (quadratic) | 111.0499 | -48.1466 | 0.5638 |
| KREC | | | | |
| 0 | Days since injury (linear) | 156.5103 | -71.9824 |  |
| 1 | Days since injury (quadratic) | 158.2131 | -71.7212 | 0.4698 |
| 2 | Days since hospitalization | 158.4488 | -72.9566 |  |
| 3 | Days since hospitalization (quadratic) | 144.55 | -64.8966 | 0.0001 |

Abbreviations: AICc, Corrected Akaike Information Criterion (lower values indicate superior model fit).

**Supplementary Table S3.** Combined Linear Mixed-Effects Model for TREC and KREC including clinical time (days since hospitalization), biological baseline (injury-to-hospitalization lag), and patient characteristics

| Parameter | Coefficient | SE | z | *p*>\|z\| | CI% [0.025, 0.975] |
| --- | --- | --- | --- | --- | --- |
| TREC | | | | | |
| Intercept | 1.953 | 0.139 | 14.087 | 0.000 | 1.681, 2.225 |
| Sex | -0.423 | 0.151 | -2.804 | 0.005 | -0.719, -0.127 |
| Age centered | -0.026 | 0.005 | -4.795 | 0.000 | -0.036, -0.015 |
| Days since hospitalization | 0.002 | 0.001 | 1.867 | 0.062 | 0.000, 0.004 |
| Lag | 0.000 | 0.001 | 0.032 | 0.974 | -0.001, 0.001 |
| Group Var | 0.133 | 0.163 |  |  |  |
| KREC | | | | | |
| Intercept | 1.947 | 0.173 | 11.282 | 0.000 | 1.609, 2.285 |
| Sex | -0.221 | 0.188 | -1.175 | 0.240 | -0.589, 0.148 |
| Age centered | -0.004 | 0.007 | -0.533 | 0.594 | -0.016, 0.009 |
| Days since hospitalization | 0.001 | 0.001 | 0.663 | 0.507 | -0.002, 0.003 |
| Lag | 0.000 | 0.001 | 0.162 | 0.871 | -0.001; 0.002 |
| Group Var | 0.209 | 0.214 |  |  |  |

Abbreviations and Legend: SE, standard error; CI, confidence interval; Group Var, Between-patient variance. Male sex was modeled using female as the reference group. The 'Lag' term represents the fixed time interval between the date of injury and the date of hospitalization for each patient.

**Supplementary Table S4.** Statistical characteristics of the baseline linear mixed-effects models for TREC and KREC (including age, sex, and days since hospitalization)

| Parameter | Coefficient | SE | *p*-Value | 95% CI |
| --- | --- | --- | --- | --- |
| Intercept | 1.8815 | 0.1395 | 0.0000 | 1.6080, 2.1550 |
| Sex | -0.3615 | 0.1541 | 0.0190 | -0.6636, -0.0595 |
| Age centered | -0.0276 | 0.0052 | 0.0000 | -0.0379, -0.0173 |
| Days since hospitalization | 0.0019 | 0.0011 | 0.0813 | -0.0002; 0.0041 |
| Group Var | 1.7464 | 0.5954 | 0.0034 | 0.5795; 2.9134 |
| TREC ICC: 64.17% (Between-patient variance: 0.1288; Within-patient variance: 0.0719) | | | | |
| KREC | | | | |
| Intercept | 1.9051 | 0.1720 | 0.0000 | 1.5679; 2.2423 |
| Sex | -0.2106 | 0.1899 | 0.2675 | -0.5829; 0.1617 |
| Age centered | -0.0053 | 0.0064 | 0.4084 | -0.0179; 0.0073 |
| Days since hospitalization | 0.0009 | 0.0013 | 0.4796 | -0.0017; 0.0036 |
| Group Var | 1.8502 | 0.6319 | 0.0034 | 0.6117; 3.0887 |
| KREC ICC: 65.95% (Between-patient variance: 0.2034; Within-patient variance: 0.1050) | | | | |

Abbreviations and Legend: SE, standard error; CI, confidence interval; Group Var, Between-patient variance; ICC, intraclass correlation coefficient. Male sex was modeled using female as the reference group.

**Supplementary Table S5.** LMM results testing the significance of the interaction between age and sex on TREC levels

| Parameter | Coefficient | SE | *p*-Value | 95% CI |
| --- | --- | --- | --- | --- |
| Intercept | 1.8785 | 0.1377 | 0.0000 | 1.6085, 2.1485 |
| Sex | -0.3615 | 0.1514 | 0.0169 | -0.6581, -0.0648 |
| Age centered | -0.0309 | 0.0121 | 0.0104 | -0.0545, -0.0073 |
| Age centered × sex | 0.0040 | 0.0133 | 0.7641 | -0.0221, 0.0301 |
| Days since hospitalization | 0.0019 | 0.0011 | 0.0794 | -0.0002, 0.0041 |
| Group Var | 1.6884 | 0.5682 | 0.0030 | 0.5747, 2.8020 |

Abbreviations and Legend: SE, standard error; CI, confidence interval; Group Var, Between-patient variance. Male sex was modeled using female as the reference group.

**Supplementary Table S6.** Model selection results using hierarchical regression (AICc-based comparison)

| Biomarker | Model specification | Included predictors | k | AICc | Delta AICc |
| --- | --- | --- | --- | --- | --- |
| TREC | Final Model | Base variables + S/I index | 4 | 80.43 | 0.00 |
|  | Extended Model | Base variables + S/I index + lym | 5 | 82.71 | +2.28 |
|  | Baseline | Age + sex + time (base) | 3 | 104.45 | +24.01 |
|  | Alternative | Base variables + lym | 4 | 106.43 | +26.00 |
| KREC | Final Model | Base variables (age + sex + time) | 3 | 149.71 | 0.00 |
|  | Alternative | Base variables + S/I index | 4 | 150.88 | +1.17 |
|  | Alternative | Base variables + lym | 4 | 151.43 | +1.72 |
|  | Extended Model | Base variables + S/I index + lym | 5 | 152.52 | +2.81 |

Abbreviations and Legend: k represents the number of fixed-effect predictors; AICc, Corrected Akaike Information Criterion (lower values indicate superior model fit); Delta AICc is the difference relative to the best-fitting model in the set. Base Variables include age (centered), sex, and days since hospitalization. Terms: S/I index, severity/inflammation index; lym, lymphocyte count (**×** 10⁹/L**)**; time, days since hospitalization.

**Supplementary Table S7.** Statistical characteristics of the final optimal multivariate model for TREC and KREC identified via hierarchical regression (including the severity/ inflammation index)

| Variable | | Coefficient | | SE | *p*-Value | 95% CI | |
| --- | --- | --- | --- | --- | --- | --- | --- |
| TREC | | | | | | | |
| Intercept | | 1.8206 | | 0.1317 | 0.0000 | 1.5625, 2.0787 | |
| Sex | | -0.2816 | | 0.1459 | 0.0535 | -0.5675, 0.0042 | |
| Age centered | | -0.0270 | | 0.0049 | 4.85E-08 | -0.0367, -0.0173 | |
| Severity/inflammation index | | -0.1025 | | 0.0187 | 3.98E-08 | -0.1391, -0.0659 | |
| Days since hospitalization | | 0.0019 | | 0.0009 | 0.0468 | 2.69E-05, 0.0037 | |
| Group Var | | 2.2914 | | 0.7598 | 0.0026 | 0.8022, 3.7807 | |
| TREC ICC: 69.79% (Between-patient variance: 0.1219; Within-patient variance: 0.0528) | | | | | | | |
| KREC | | | | | | | |
| Intercept | 1.9526 | | 0.1725 | | 0.0000 | | 1.6146, 2.2906 |
| Sex | -0.2133 | | 0.1902 | | 0.2622 | | -0.5862, 0.1596 |
| Age centered | -0.0029 | | 0.0066 | | 0.6640 | | -0.0158, 0.0101 |
| Days since hospitalization | 0.0009 | | 0.0013 | | 0.5014 | | -0.0017, 0.0035 |
| Group Var | 2.1559 | | 0.7020 | | 0.0021 | | 0.7800, 3.5318 |
| KREC ICC: 67.17% (Between-patient variance: 0.2110; Within-patient variance: 0.1031) | | | | | | | |

Abbreviations and Legend: SE, standard error; CI, confidence interval; Group Var, Between-patient variance; ICC, intraclass correlation coefficient. Male sex was modeled using female as the reference group.

**Supplementary Table S8.** Exploratory interaction analysis of TREC recovery dynamics

| Parameter | Coefficient | *p*-value |
| --- | --- | --- |
| Intercept | 1.8035 | 2.38E-43 |
| Sex | -0.2616 | 0.0702 |
| Age centered | -0.0270 | 2.25E-08 |
| Days since hospitalization | 0.0017 | 0.0652 |
| Severity/inflammation index | -0.1478 | 1.67E-05 |
| Days since hospitalization **×** severity/inflammation index | 0.0009 | 0.1179 |
| Group Var | 2.3739 | 0.0024 |

Abbreviations and Legend: Group Var, Between-patient variance. Male sex was modeled using female as the reference group.

**Supplementary Table S9.** Bootstrap robustness results for TREC and KREC: Validation of the final TREC model (1,000 iterations)

| Parameter | Original Coeff. | Boot Mean | Bias | Boot SE | 95% Bootstrap CI |
| --- | --- | --- | --- | --- | --- |
| TREC | | | | | |
| Intercept | 1.8207 | 1.8148 | -0.0059 | 0.1083 | 1.6263, 2.0353 |
| Sex | -0.2868 | -0.2794 | 0.0074 | 0.1255 | -0.5267, -0.0505 |
| Age centered | -0.0271 | -0.0272 | -0.0001 | 0.0050 | -0.0373, -0.0174 |
| Severity/inflammation index | -0.1024 | -0.1122 | -0.0098 | 0.0256 | -0.1687, -0.0765 |
| Days since hospitalization | 0.0019 | 0.0017 | -0.0001 | 0.0010 | -0.0003, 0.0038 |
| KREC | | | | | |
| Intercept | 1.9520 | 1.9502 | -0.0018 | 0.1191 | 1.7149, 2.1835 |
| Sex | -0.2130 | -0.2066 | 0.0064 | 0.1511 | -0.5081, 0.1008 |
| Age centered | -0.0029 | -0.0028 | 0.0001 | 0.0062 | -0.0135, 0.0107 |
| Days since hospitalization | 0.0009 | 0.0006 | -0.0003 | 0.0016 | -0.0032, 0.0029 |

Abbreviations and Legend: SE, standard error; CI, confidence interval. Male sex was modeled using female as the reference group.

**Supplementary Table S10.** TREC model bootstrap correlation matrix of estimates

|  | Intercept | Sex | Age centered | S/I index | Time | Group Var |
| --- | --- | --- | --- | --- | --- | --- |
| Intercept | 1 | -0.8395 | 0.0938 | 0.0911 | -0.0094 | 0.0378 |
| Sex | -0.8395 | 1 | 0.1477 | -0.2082 | -0.0469 | 0.0308 |
| Age centered | 0.0938 | 0.1477 | 1 | 0.0635 | 0.1545 | 0.4938 |
| S/I index | 0.0911 | -0.2082 | 0.0635 | 1 | 0.3922 | 0.0690 |
| Time | -0.0094 | -0.0469 | 0.1545 | 0.3922 | 1 | 0.1544 |
| Group Var | 0.0378 | 0.0308 | 0.4938 | 0.0690 | 0.1544 | 1 |

Abbreviations and Legend: S/I index, severity/inflammation index; Time, days since hospitalization; Group Var, Between-patient variance. Male sex was modeled using female as the reference group.


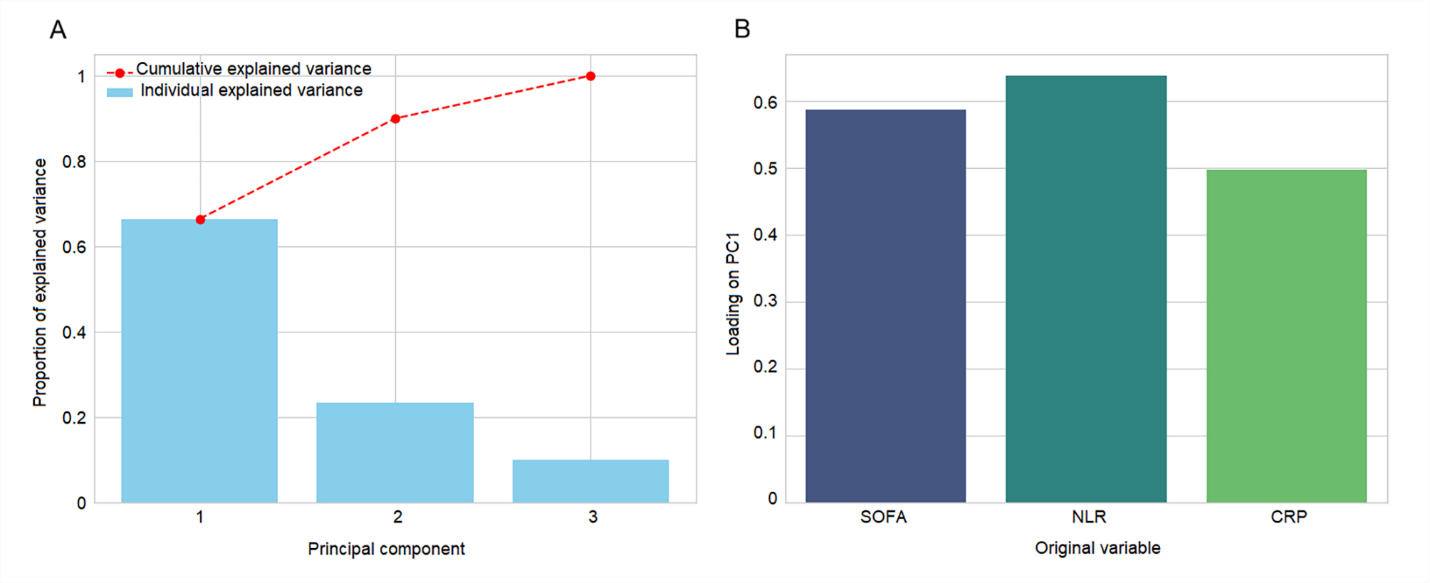


**Supplementary Figure S1**. Principal Component Analysis (PCA) of clinical markers. (**A**) Scree plot showing the proportion of total variance explained by each principal component. The first component (PC1) accounts for 66.4% of the variance, and the steep drop-off justifies its use as a summary measure. (**B**) Loadings plot illustrating the contribution of each original variable (SOFA, NLR, CRP) to PC1. The positive and strong loadings for all three variables confirm that PC1 serves as a unified 'severity/inflammation index'. Abbreviations: SOFA, Sequential Organ Failure Assessment; NLR, Neutrophil-Lymphocyte Ratio; CRP, C-reactive protein.


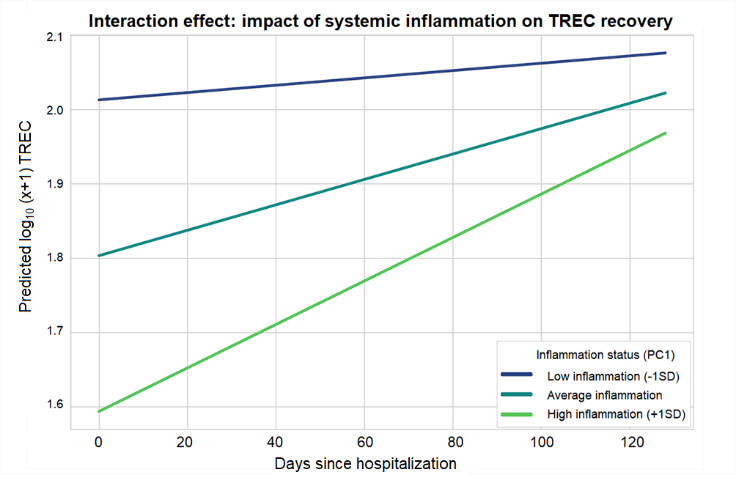


**Supplementary Figure S2**. Predictive margins plot of the interaction between inflammation and TREC recovery. Thick colored lines represent the predicted marginal mean trajectories for TREC levels across the hospitalization period, derived from the interaction model. Conditions: predictions are stratified by the severity/inflammation index (PC1) at three levels: low inflammation (-1 SD, blue), average inflammation (mean, blue-green), and high inflammation (+1 SD, green). Abbreviations: SD, Standard Deviation.

**
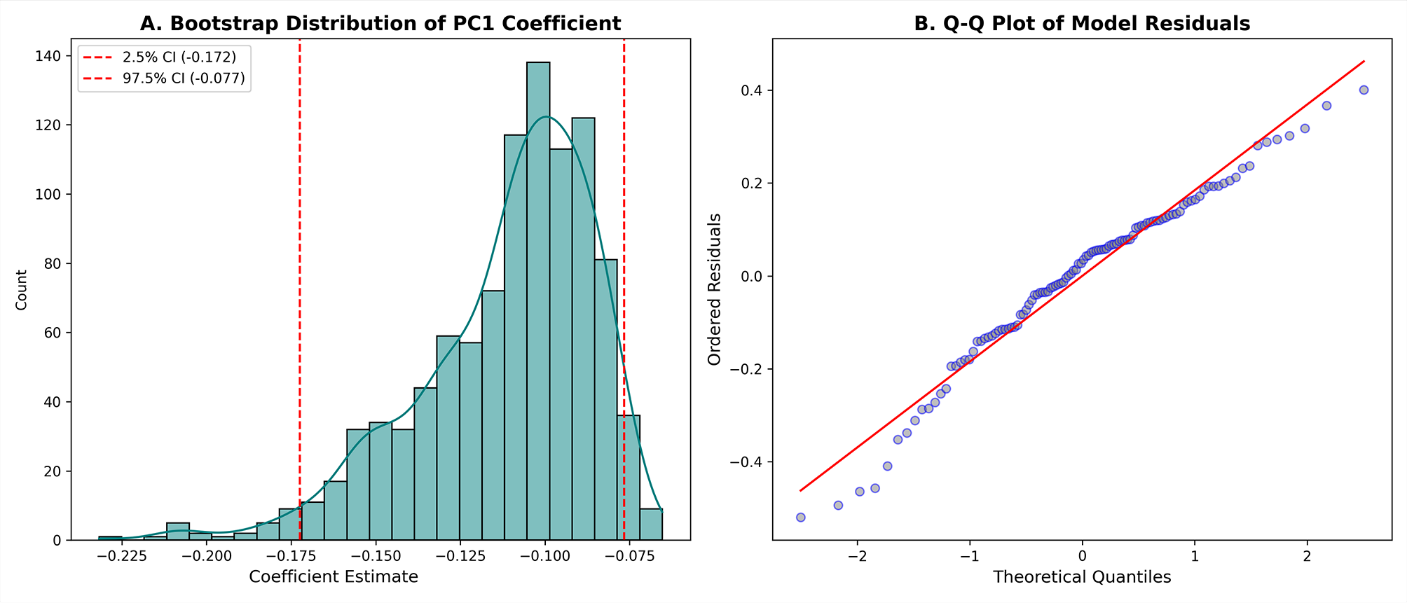
**

**Supplementary Figure S3.** Visualization of bootstrap distribution and normality assessment for the severity/inflammation index (PC1). (**A**) Bootstrap distribution of the PC1 coefficient, including its 95% confidence interval. (**B**) Bootstrap Q-Q plot of residuals, indicating a non-normal distribution. Abbreviations: Q-Q plot, Quantile-Quantile plot; CI, confidence interval.

**Supplementary References**

1. Cho, S, Ge, J, Seo, SB, Kim, K, Lee, HY, and Lee, SD. Age Estimation via Quantification of Signal-Joint T Cell Receptor Excision Circles in Koreans. *Leg Med.* (2014) 16(3):135–138. doi: 10.1016/j.legalmed.2014.01.009
2. Lorenzi, AR, Patterson, AM, Pratt, A, Jefferson, M, Chapman, CE, Ponchel, F, Isaacs, and JD. Determination of thymic function directly from peripheral blood: a validated modification to an established method. *J Immunol Methods*. (2008) 339(2):185–94. doi: 10.1016/j.jim.2008.09.013
3. Ou, X, Gao, J, Wang, H, Wang, H, Lu, H, and Sun, H. Predicting Human Age with Bloodstains by sjTREC Quantification. *PLoS ONE*. (2012) 7:e42412. doi: 10.1371/journal.pone.0042412
4. Yamanoi, E, Uchiyama, S, Sakurada, M, and Ueno, Y. sjTREC Quantification Using SYBR Quantitative PCR for Age Estimation of Bloodstains in a Japanese Population. *Leg Med*. (2018) 32:71–74. doi: 10.1016/j.legalmed.2018.03.003
5. Kashatnikova, DA, Khadzhieva, MB, Kolobkov, DS, Belopolskaya, OB, Smelaya, TV, Gracheva, AS, et al. Pneumonia and related conditions in critically ill patients—insights from basic and experimental studies. *Int J Mol Sci*. (2022) 23(17):9896. doi: 10.3390/ijms23179896
